# Repetitive reaching training combined with transcranial Random Noise Stimulation in stroke survivors with chronic and severe arm paresis is feasible: a pilot, triple-blind, randomised case series

**DOI:** 10.1186/s12984-017-0253-y

**Published:** 2017-05-30

**Authors:** Kathryn S. Hayward, Sandra G. Brauer, Kathy L. Ruddy, David Lloyd, Richard G. Carson

**Affiliations:** 1 Division of Physiotherapy, School of Health and Rehabilitation Sciences, University of Queensland, Brisbane, Australia; 2 Department of Physical Therapy, University of British Columbia, Vancouver, Canada; 3 Department of Health Sciences and Technology, Neural Control of Movement Lab, ETH Zurich, Zurich, Switzerland; 4 Queensland Brain Institute, University of Queensland, Brisbane, Australia; 5 Trinity College Institute of Neuroscience and School of Psychology, Trinity College, Dublin, Ireland; 6 School of Psychology, Queens University Belfast, Belfast, UK

**Keywords:** Stroke, Upper limb, Non-invasive brain stimulation, Magnetic resonance imaging, Function

## Abstract

**Background:**

Therapy that combines repetitive training with non-invasive brain stimulation is a potential avenue to enhance upper limb recovery after stroke. This study aimed to investigate the feasibility of transcranial Random Noise Stimulation (tRNS), timed to coincide with the generation of voluntary motor commands, during reaching training.

**Methods:**

A triple-blind pilot RCT was completed. Four stroke survivors with chronic (6-months to 5-years) and severe arm paresis, not taking any medications that had the potential to alter cortical excitability, and no contraindications to tRNS or MRI were recruited. Participants were randomly allocated to 12 sessions of reaching training over 4-weeks with active or sham tRNS delivered over the lesioned hemisphere motor representation. tRNS was triggered to coincide with a voluntary movement attempt, ceasing after 5-s. At this point, peripheral nerve stimulation enabled full range reaching. To determine feasibility, we considered adverse events, training outcomes, clinical outcomes, corticospinal tract (CST) structural integrity, and reflections on training through in-depth interviews from each individual case.

**Results:**

Two participants received active and two sham tRNS. There were no adverse events. All training sessions were completed, repetitive practice performed and clinically relevant improvements across motor outcomes demonstrated. The amount of improvement varied across individuals and appeared to be independent of group allocation and CST integrity.

**Conclusion:**

Reaching training that includes tRNS timed to coincide with generation of voluntary motor commands is feasible. Clinical improvements were possible even in the most severely affected individuals as evidenced by CST integrity.

**Trial registration:**

This study was registered on the Australian and New Zealand Clinical Trials Registry (ANZCTR) http://www.ANZCTR.org.au/ACTRN12614000952640.aspx. Registration date 4 September 2014, first participant date 9 September 2014.

## Background

It is estimated that 30% of stroke survivors have severe upper limb impairment [1], whereby the functional capacity of the paretic arm is diminished to the extent that it cannot be moved against gravity [2]. For these individuals, who do not have sufficient movement with which to work, the provision of effective therapy can be challenging. The associated consequences are poor prospects for recovery [3], limited rehabilitation opportunities [4], and ultimately reduced quality of life (QoL) [5]. Yet, if task-oriented practice can be made possible by some means, there exists the potential to promote motor recovery, and in turn make a significant positive impact upon individual QoL and alleviate burden of care. In seeking to achieve levels of task-oriented practice beyond those that are possible through traditional therapy alone, attention has therefore turned to enabling technologies, including “assistive” devices, and adjuvant methods such as peripheral nerve and brain stimulation.

Best evidence syntheses [6, 7] suggest that goal-directed movements can be assisted by minimizing the mechanical degrees of freedom to be controlled, in combination with the augmentation of voluntary muscle activity via peripheral nerve stimulation of target muscles, or the use of mechanical actuators. To encourage positive changes in motor performance, the capacity to increase task difficulty through small, yet incremental progressions and provision of meaningful real-time visual and auditory feedback have also been highlighted [8, 9]. The authors have previously sought to implement these principles, using the Sensorimotor Active Rehabilitation Training of the Arm (SMART Arm) device to promote functional recovery in severely impaired stroke survivors [8–10]. It has been shown that 4-weeks (12-h) of community-based training of reaching in people greater than 6-months post stroke improved upper limb function (and increased reaching distance) [8], enhanced the specificity of muscle recruitment (elevated ratio of biceps to triceps activation during reaching) [11], and accentuated corticospinal reactivity (decreased motor evoked potential [MEP] onset latency) [12]. Of particular interest in the context of the current study is the observation that not all individuals achieved functional gains. In these cases, the intrinsic neurobiological reserve of the injured brain may have been insufficient for repetitive training alone to drive recovery of motor function.

A variety of non-invasive brain stimulation (NIBS) techniques are now being used with the aim of altering the excitability of brain networks that have the potential to be engaged during the execution of motor tasks. The most commonly applied NIBS techniques are transcranial-direct current stimulation (tDCS) and repetitive-transcranial magnetic stimulation (rTMS) [13]. In general, the application of these techniques is predicated on the assumption that by altering the state of circuits within (contralateral) primary motor cortex (M1) in a manner that produces *sustained* increases in the excitability of corticospinal projections to the impaired limb (or by decreasing the excitability of circuits in the M1 ipsilateral to the impaired limb), therapeutic gains will be realised. The fact that these approaches have limited efficacy in severely impaired stroke survivors notwithstanding [14], there exist other forms of therapeutic NIBS that are motivated by a different premise.

It is well established that in some circumstances, the addition of random interference or noise, enhances the detection of weak stimuli, or the information content of a signal (e.g., trains of action potentials) [15]. In light of this phenomenon, it has been proposed that the application of transcranial random noise stimulation (tRNS) may boost the adaptive potential of cortical tissue [16]. The present investigation is motivated by the conjecture that: if the delivery of random noise stimulation is timed to occur simultaneously with the generation of voluntary motor commands, it may serve to amplify functional adaptations invoked by the intrinsic neural activity.

Implemented through a triple-blind pilot randomised control design, the specific aim of this study was to establish the feasibility of delivering tRNS, timed to coincide with the generation of the voluntary motor commands, in the context of reaching movements performed by individuals with chronic and severe upper limb paresis after stroke. Recognising that the response to any therapeutic intervention is constrained by the state of pathways that can convey signals from the brain to the periphery, diffusion-weighted magnetic resonance imaging (DW-MRI) was performed to characterize the structural integrity of the descending corticospinal tract (CST) projections for each participant.

## Methods

### Design

A pilot, triple-blind, randomised case series to explore the feasibility of combining tRNS with reaching training was conducted between September and December 2014 in the Department of Physiotherapy at the University of Queensland, Brisbane Australia. Ethical approval was received from the University of Queensland Medical Research Ethics Committee (2014000263). All participants provided written informed consent to participate and have their findings published in accordance with the Declaration of Helsinki. This study was registered on the Australian and New Zealand Clinical Trials Registry (ANZCTR) http://www.ANZCTR.org.au/ACTRN12614000952640.aspx.

### Participants

Four stroke survivors were recruited using two methods. Firstly, we contacted people in our research group database that had consented to be contacted for stroke research and resided in Brisbane Australia. Secondly, we posted recruitment flyers with the following sources: a) National Stroke Foundation of Australia webpage; b) Queensland Rehabilitation Physiotherapy Network; and c) physiotherapy and speech pathology clinics at the University of Queensland. Eligible participants were first time stroke survivors who were between 6-months and 5-years post stroke, aged over 18 years, and presented with severe upper limb paresis (as indicated by a triceps manual muscle test score of 1+, 2- or 2 out of 5, and Motor Assessment Scale item 6 score of <4 out of a possible 6 points). In addition, all participants were required to be able to understand single stage commands, and have not participated in any upper limb related therapy with a therapy service for at least 2-weeks prior to baseline assessment. Exclusion criteria were: 1) any contraindications to tRNS (e.g., history of seizures) or MRI (e.g., pacemaker); 2) presence of any neurological condition other than stroke (e.g., Parkinson’s disease), 3) elbow contracture greater than 15°, or 4) consumption of medication/s that could alter cortical excitability (e.g., antiepileptic medications, antidepressants) or have a presumed positive or negative effect on neural plasticity (e.g., dopamine, dexamphetamine). The general practitioner of each participant was contacted to provide a list of current medications that was reviewed by a clinical pharmacist to determine known or presumed effect on neural plasticity.

### Blinding and randomization

This was a triple blind study. Study personnel involved in assessment and training, along with all participants were blinded to group allocation for the duration of the study. An offsite investigator prepared the concealed randomization using a computer generated random number sequence (1:1). Groups were: 1) active-tRNS + reaching training or 2) sham-tRNS + reaching training. Consecutively numbered opaque envelopes containing group allocation were collected from the offsite investigator by the tRNS intervener after initial assessment. The blinding code was shared with the investigator team on completion of training and follow-up assessments of all four participants.

### Intervention

**Fig. 1:**
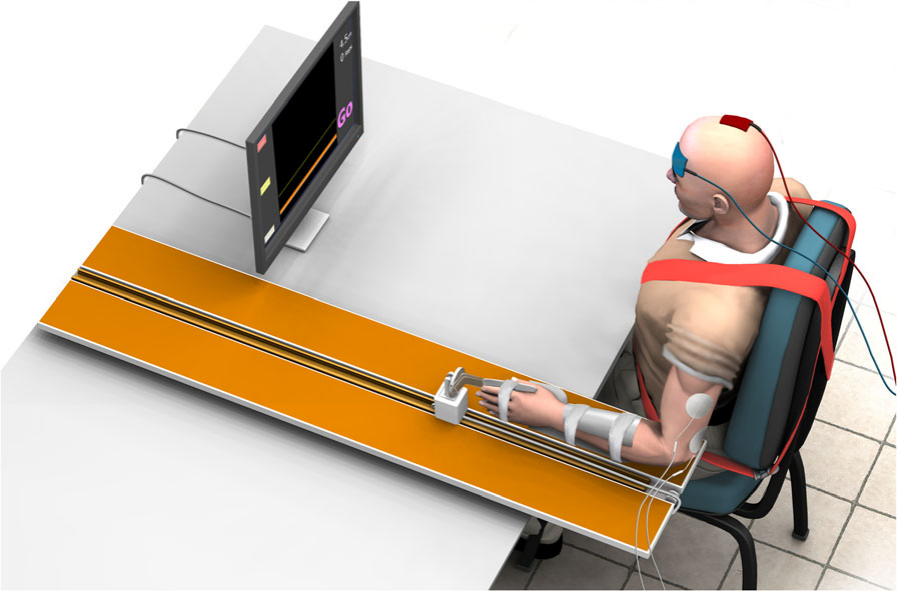
Representation of the training setup including horizontal reaching track, trunk restraint, visual feedback, transcranial random noise stimulation application, and electrical stimulation application to lateral head of triceps

Participants underwent 12 reaching training sessions of 45-m duration over 4-weeks (9 h total) in the Neurological Ageing and Balance Research Unit at the University of Queensland. Set up and pack up time was separate from training time. During training, participants were encouraged to perform as many repetitions as possible within time allocation. The training set-up and protocol replicated that previously established to produce a statistically and clinically meaningful change in upper arm function in chronic stroke survivors [8]. The set-up is visually depicted in Fig. 1. To augment full range reaching and enable independent practice, reaching training included outcome-triggered electrical stimulation (OT-stim) that was delivered up to the point at which the individual surpassed their personal best reaching distance [9]. The difficulty of training was incrementally progressed to ensure training was challenging through increased number of repetitions, reduced rest time, track elevation and addition of load. All reaching training was recorded in a training log.

tRNS was delivered by a battery-driven electrical stimulator (Magstim, UK) through conductive rubber electrodes, placed in saline soaked sponge sleeves. For both active-tRNS and sham-tRNS, the electrodes were positioned as per the 10/20 international system for C3/C4 EEG electrode placement [17]: the stimulation electrode was positioned over the ipsilesional primary motor cortex (M1), and the reference electrode was positioned over the contralateral supra-orbital region. The current was set to 2 mA for active-tRNS and 0 mA for sham-tRNS, for 5 s duration and 0 s fade in/out. The tRNS intervener informed participants that they may or may not feel the stimulation irrespective of whether they were receiving active or sham stimulation. All tRNS parameters were recorded by the independent tRNS intervener in a separate log that was stored in a separate locked filing cabinet to that containing training and assessment information.

The delivery of tRNS was timed to coincide with voluntary motor drive. During reaching training, visual (‘go’ on computer screen) and auditory (beep) signals were used to indicate to the participant that they were to commence their reaching attempt. The delivery of tRNS was programmed to commence simultaneously with the onset of these visual and auditory signals, and to cease after 5-s – at which point the voluntary reach movement attempt was generally still in progress. When required (personal best reaching distance less than passive reaching distance), OT-stim to the impaired lateral head of triceps brachii muscle was triggered to augment voluntary movement to ensure that full range reaching was achieved.

### Measures

Participants were assessed at baseline (pre-training, 0-weeks), post-training (4-weeks) and follow-up (12-weeks) in the Neurological Ageing and Balance Research Unit at the University of Queensland. Personal and stroke-related details were collected and the modified Rankin Scale performed to describe the severity of disability present at baseline. Adverse events were reported in the training log (e.g., fall, negative response to tRNS or OT-stim) during training, and the follow-up assessment book for post training events (e.g., fall). Training outcomes of sessions and repetitions completed were recorded in training logs.

#### Clinical outcomes

All clinical measures were obtained at each timepoint. The primary clinical outcome measure was Motor Assessment Scale item 6 (Upper Arm Function, MAS6) [18]. Secondary outcome measures were MAS item 7 (hand activities) and item 8 (advanced hand activities), along with impairment measures of muscle strength of triceps brachii and extensor carpi radialis, resistance to passive movement (Modified Ashworth Scale) and spasticity (Tardieu Scale) of elbow and wrist flexion [19], and shoulder pain on passive external rotation (Ritchie Articular Index) [20]. In addition, upper limb participation was evaluated according to the Rating of Everyday Arm Use in the Community and Home (REACH) scale [21].

#### MRI

MRIs were acquired pre and post training at the Centre for Advanced Imaging at The University of Queensland on a Siemens Magnetron 3T Trio whole body scanner using a 32-channel encoding head coil. A high-resolution T1-weighted anatomical scan (0.45 x 0.45 x 0.9 mm voxel size) was collected to determine lesion location. A single high-angular resolution diffusion imaging (HARDI) scan was subsequently performed using a single shot echo-planar imaging (EPI) sequence (TR = 9000 ms, TE = 113 ms, FOV = 230 x 230 mm, 55 slices, voxel dimensions = 2.3 x 2.3 x 2.3 mm^3^). Diffusion weighting was applied across 85 independent non-collinear orientations (b = 3000 s/mm^2^), along with three un-weighted images (b = 0 s/mm^2^). Only pre-training MRIs were evaluated.

#### Post-training interview

In depth, one-on-one interviews were completed with each stroke survivor and their main carer (in the case of aphasic participants) to encourage comfortable sharing of personal information and individual experiences, and to facilitate individualized probing. Interviews were arranged by the blinded assessor and conducted by a facilitator not involved in any aspect of the study, who was independent of the research institution and therapy services the participant had engaged in.

Four open-ended stimulus questions (Table 1) underpinned each interview. In these discussions, the term function was used to encapsulate the ICF domains of impairment, activity and participation. At the commencement of each discussion, the facilitator explained the interview purpose and then proceeded to ask each of the four key questions. There was no strict adherence to the style and type of questioning beyond these four questions, with probes used to explore or challenge emerging themes, personal experiences and ideas. All discussions were drawn to a close with the facilitator summarizing the main points raised. The participants were then provided with the opportunity to add or dispute what had been said or contribute any final thoughts.

**Table 1:** Primary question(s) within each category of questing for in-depth interviews

(1) Understanding of upper limb rehabilitation processes:
*“Prior to commencing this study what was your understanding of upper limb therapy? How does this differ now that you’ve undertaken this research project of reaching training?”*
(2) Reaching training:
*“Thinking about the reaching training, can you tell me how you felt doing the training?”*
(3) Problems as well as rewarding situations during your reaching training:
*“Was there anything that stopped you from wanting to/helped you continue with the research project? If so, can you tell me about this?”*
(4) Advice you might have about upper limb rehabilitation:
*“Do you think that this type of training helped your arm recover? Why/why not? What would you say to someone about to commence this type of reaching training? What would you want them to know?”*

### Analysis

To determine feasibility, we considered adverse events, training outcomes, clinical outcomes, structural integrity of descending motor projections, and reflections on training through in-depth interviews from each individual case.

#### Adverse events

Number of adverse events recorded during training (e.g., complaints of pain, discomfort) and at follow up assessment (e.g., falls).

#### Training outcomes

Number of sessions attended and missed were tallied and average repetitions per session (total repetitions/number of sessions) were calculated for each individual.

#### Clinical outcomes

Change from pre to post intervention for the primary and secondary outcome measures were calculated for each individual. A clinically meaningful change was considered to be a 10%, or a 1-point change on MAS6. This is consistent with previous work conducted by our group [9, 22] and others [23].

#### Structural integrity of descending motor projections

The high resolution T1 anatomical scans acquired in the ‘pre’ sessions were used for transformation of the diffusion weighted imaging (DWI) data in ExploreDTI (Leemans et al 2009) so that both image modalities (displaying white matter and grey matter together) could be co-registered and superimposed to facilitate identification of anatomical structures in the brain. DWI data were first visually inspected for excessive motion artifact or instrumental noise using quality assurance tools available in the diffusion MRI software package ExploreDTI v.4.8.4 [24]. For all images, signal intensity was modulated and the b-matrix rotated [25]. Imaging data were then corrected for motion and distortion. As there were only four participants, it was possible to use a manual ‘region of interest’ identification procedure, whereby the white matter tracts of the CST in the posterior limb of the internal capsule were extracted by hand-drawing around the anatomical region on the superimposed DWI-T1 native images. Constrained spherical deconvolution (CSD) was used to model diffusion behavior [26] as it is robust in the presence of crossing fibre populations [27]. Crossing fibres are estimated to occur in greater than 90% of white matter voxels in the brain [28]. Additionally, CSD does not make assumptions regarding uniform diffusion of water within a voxel [26, 29] and is more sensitive in the severely damaged brain [27]. CSD-based deterministic whole-brain fibre tractography was initiated at each voxel using the following parameters: seed-point resolution of 2 mm^3^, 0.2 mm step size, maximum turning angle of <40°, and fibre length range of 50–500 mm [30]. Tractography employed a fibre alignment by continuous tracking algorithm approach [31] with Fractional Anisotropy (FA) values extracted from reconstructed streamlines. Fractional Anisotropy is a quantitative, unit-less measure of diffusion behaviour of water in the brain influenced by microstructural properties of white matter and is the most commonly reported measure of white matter microstructural properties after stroke [32]. Having extracted CST FA for both the lesioned and non-lesioned hemispheres for each participant, a FA ‘asymmetry index’ (AI) was calculated according to Eq. 1. This index quantifies the degree of degeneration of white matter in the tracts that are responsible for conveying motor cortical commands to the muscles of the upper limb. For our purposes, an asymmetry index of 0.01-0.05 was considered mild, 0.06-0.15 was considered moderate, and >0.15 was considered severe. As the AI increases, greater loss of white matter structural integrity as a result of the stroke can be inferred.

1 $$ \begin{array}{c}\hfill \mathrm{AI} = \kern0.75em \left(\mathrm{FA}\ \mathrm{non}\hbox{-} \mathrm{lesioned}\ \mathrm{PLIC}\ \hbox{--}\ \mathrm{FA}\ \mathrm{lesioned}\ \mathrm{PLIC}\right)\hfill \\ {}\hfill \left(\mathrm{FA}\ \mathrm{non}\hbox{-} \mathrm{lesioned}\ \mathrm{PLIC} + \mathrm{FA}\ \mathrm{lesioned}\ \mathrm{PLIC}\right)\hfill \end{array} $$

#### Reflections on training

All audio recordings of the in-depth interviews were transcribed verbatim and cross-checked by another researcher against the audio record to verify accuracy. An approach consistent with conventional thematic content analysis was used [33]. On completion of the study, the transcripts of each participant were explored independently through a process of reading and re-reading. Two researchers, one of whom was involved in training (KH) and one who was not involved in any data collection procedures (SB), independently reviewed all transcripts. On the first reading, transcripts were read in their entirety to acquire a whole sense of the data. On the second reading, line-by-line analysis was used to identify themes, patterns or concepts. This led to the tentative collation of predominant themes emerging across participants. The two reviewers met at this point and discussed their themes, looking for patterns or concepts that were both consistent and inconsistent with each other. Consensus themes were identified. A final reading of the data was used to check the fit of the consensus themes with the transcripts, pursuing patterns or concepts that were both consistent and inconsistent with the data. A second meeting of the reviewers occurred to confirm the themes, or modify them as required to more appropriately represent the data. At this point, the conditions under which each theme arose and its relationship to other themes (within and between groups) were documented. All findings are anonymised.

## Results

Four participants were recruited, with maximum variation in sample achieved (See Table 2), Each participant was treated as a single case.

**Table 2:** Participant characteristics

ID	Age at training	Stroke type	MSS	Dominant arm	Paretic arm	Aphasia	Baseline mobility, FAC,/6	mRS,/5
P1	49	Ischaemic	24	Right	Left	No	6	3
P2	53	Ischaemic	32	Right	Right	Yes	5	3
P3	73	Ischaemic	25	Right	Left	No	1	5
P4	70	Ischaemic	37	Right	Right	Yes	4	3

*FAC* Functional Ambulation Category, *MSS* months since stroke, *mRS* modified Rankin Scale

### Adverse events

There were no adverse events recorded during the course of training, and no adverse events were reported to have occurred during follow up.

### Training outcomes

All four participants completed the 12 training sessions over 4-weeks, with no training sessions missed. Participants completed on average 117 reaching repetitions per session. Participant 1 completed the most repetitions per session on average (*n* = 147), while participant 3 completed the fewest repetitions on average per session (*n* = 85).

### Clinical outcomes

Participants (P01 and P02) demonstrated an improvement in triceps muscle strength (impairment), and REACH Scale (participation) post training, which was maintained at follow-up. P02 also demonstrated an improvement in MAS6 (activity) post training, which was lost at follow up. P03 demonstrated an improvement in MAS6 (activity), which was maintained at follow up. P04 demonstrated an improvement in triceps muscle strength (impairment) post training that was maintained at follow up. Interestingly, both P03 and P04 demonstrated no change in use of the arm in everyday tasks (i.e., participation) post training, however they did demonstrate an improvement in this measure at follow up. See Table 3.

**Table 3:** Training, clinical and descending motor projection outcomes

ID	tRNS group	Total repetitions	Wrist MRC/15			Triceps MRC/15			MAS6,/6			REACH,/5			Asymmetry index
			T0	T1	T2	T0	T1	T2	T0	T1	T2	T0	T1	T2	
P1	Active	1763	2	3	3	10	12	12	1	1	1	1	2	2	0.02
P2	Placebo	1128	2	2	2	3	6	7	2	3	2	0	1	1	0.11
P3	Active	1015	0	0	1	3	3	2	0	1	1	0	0	1	NP
P4	Placebo	1696	1	1	2	3	6	6	1	1	1	0	0	1	NP

*MAS6* Motor Assessment Scale Item 6 Upper Arm Function, *MRC* Medical Research Council strength grading including + and – to achieve a possible 15 points, *NP* not possible, *REACH* Rating Everyday Arm use in the Community and Home, *tRNS* transcranial random noise stimulation; *T0* baseline (0-weeks), *T1* post training (4-weeks), *T2* follow up (12-weeks)

### Structural integrity of descending motor projections

**Fig. 2:**
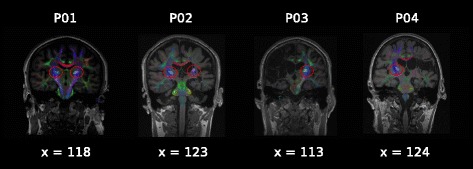
Corticospinal tract streamline reconstructions: the corticospinal tract is indicated for each of the four participants, displayed on coronal (x view) slices of T1 weighted anatomical scans with direction encoded fractional anisotropy (FA) colour maps superimposed. Images are shown in radiological format (ie. right on the image is the patient’s left side). The reconstructed streamlines for the corticospinal tract are also superimposed, and indicated by red circles. The posterior limb of the internal capsule (PLIC) within the corticospinal tract was the region of interest that was delineated manually for each scan, using anatomical landmarks. No tracts were detected in the PLIC region in the right hemisphere for P03, or the left hemisphere for P04

Participant P01 (0.02) had a mild AI, P02 (0.11) had a moderate AI, and P03 and P04 had a severe AI (See Table 3). This suggests that P01 and P02 had residual CST reserve, and thus perhaps some potential for UL recovery, but were yet to realize this potential. In contrast, P03 and P04 had such extensive structural loss of white matter in the CST, that no streamlines could be reconstructed in this region of the ipsilesional hemisphere. Visualizations of the CST for each individual are displayed in Fig. 2.

### Reflections on training

Overall participants and their carers (where involved) spoke positively about the training program with active or sham tRNS, describing it as feasible and beneficial. No participants described any adverse effects of the stimulation, nor was it perceived to interfere with engagement in training. One participant even described it as “*trying to say hey come on, let’s keep moving,* P01”. The only negative of training, described by all, was that training was ending – expressing that they wanted to be able to continue to participate due to the gains experienced. “*Now that it has stopped … we’d like it to keep going … if you could extend it, it would be even more valuable*, P04 carer”. Beyond these general comments, the content of the interviews could be ascribed to three themes. Stroke survivors and their carers described the *individualised training* helped them to *maintain motivation* and fostered the overwhelming sense that *I haven’t lost all my chances of improving my arm*. Each theme will be discussed in turn.

***Individualised training*** was described as a positive aspect of this combination therapy approach. Participants described it as not just a set program, but rather an individualised training package that included coaching, education and meaningful repetition. The coaching skills of the trainer were described as critical to training engagement. Coaching included tailoring training in a way that “*forces you to move and achieve a goal*, P02”. In this way it was thought that a better quality of movement was achieved. While people felt they could do training alone, “*what really helped was having someone beside me, pushing you on and motivating you*, P01”. A strong component of individualization expressed by participants was education, described as “*direction and guidance on what to do and how to do it*, P02 carer”. Many commented that previously they did not receive such guidance, which made it challenging to maintain concentration during training. Engagement in meaningful repetition performed within a combination-training program was viewed positively. *“This training with the stimulation and the movement and actually showing us how it is happening, and then doing it with repetition. It clicked,* P02 carer” and “*I knew I was doing a defined task … and was trying to improve each time … it was good*, *P01”*.

Individuals described that ***maintaining motivation*** was important throughout training. All described that during the first week of training, little gains were experienced and recovery was slow. Throughout this period hope that training might trigger a change in one’s recovery pathway was important. “*It’s only going to happen if you keep working on it,* P03” and “*she has the determination to concentrate on the arm,* P04 carer”. Maintaining motivation was challenged by not being able to see the achievements that were happening. “*It can be frustrating. We like to see everything visually represented as achievement. But some of the achievements that are happening are quietly occurring in our brain so when the actual product will happen might take a few weeks. I think patience is very important,* P02 carer”. But with patience and the ability to just hang in there, training was described to eventually “*just sort of click*, P01”. Individuals described taking ownership of performance-related changes within a session, small or large, to positively reinforce engagement in use of the arm outside of therapy – an important shift in mindset that contributed to maintained motivation.

It was overwhelmingly evident that a major benefit of the training was realizing that “***I (stroke survivor) haven’t lost all my chances of improving my arm***”. Many described recovery of the arm early after stroke as “*forgotten and neglected*, P01”. Realising that engagement in arm training was possible, many described a renewed hope for recovery. Statements such as “*made me feel like there is hope there for moving my (his) arm*, *P01 and P03 carer*” were common. Carers described a sense of relief, that the hope they had sustained in seeing an improvement in their loved ones arm function was not hopeless. *“We believed there was a way to do it, but just didn't know what the way was, so it was like oh yes, it is possible,* P02 carer*”.*

## Discussion

This study demonstrated that gains in relation to both impairment and function are possible in the chronic phase of recovery in people with severe upper limb impairment – even in those with limited neurobiological structural reserve for recovery. While all participants improved, the nature of the improvement was specific to each individual. Despite demonstrating that tRNS timed to coincide with the generation of the voluntary motor commands during reaching training was feasible, it was not evident in this very small sample, that there was a benefit over and above reaching training alone (sham tRNS). Given all participants enjoyed the training, perceived it to be beneficial and wanted it to continue, there is impetus to explore this training paradigm more extensively.

Few studies to date have sought to document the condition of the motor system prior to engaging individuals (with severe impairment) in therapeutic training. Without first defining the state of pathways that convey signals from the brain – operationalized in the present instance as the structural integrity of the corticospinal tract – it is challenging to determine the extent to which this acts as a constraint on the gains that can be achieved as a result of an intervention. In the present study all four individuals had clinically severe upper limb impairment and demonstrated treatment related gains. Yet, they variously exhibited mild, moderate, and severe degeneration of the white matter tracts that pass through the posterior limb of the internal capsule. It appears therefore that in this study, the gains realised through training were not manifestly constrained by the integrity of corticospinal projections to the affected limb. This is consistent with a previous study using a similar training intervention [12], whereby some individuals who failed to exhibit a MEP at the outset, nonetheless accrued benefit from training. In that case, the functional integrity of the corticospinal tract was inferred from the presence or absence of MEPs invoked by TMS in a principal agonist (triceps brachii). Taken together, these outcomes suggest that for chronic stroke survivors who present with severe impairment of motor function, the integrity of corticospinal projections – at least as contemporary DWI and TMS methodologies assess them, does not exert a determining influence on the gains that can be realised through repetitive reach training.

The present study is unique in the field of stroke rehabilitation as it combines assistive therapy, with peripheral and cortical stimulation techniques. Two key aspects separate our work from that conducted previously: 1) cortical stimulation was timed to coincide with the preparation and production of active movement, and this was followed up with 2) peripheral stimulation that augmented voluntary motor output to enable task completion. It remains to be seen however, if this paradigm can enhance recovery over and above that achieved with a particular intervention in isolation. This is a common challenge for stroke rehabilitation and recovery research, particularly in relation to the application of NIBS [34]. Nonetheless, the approach we have described appears feasible and was also received favourably by the stroke survivors and carers involved.

### Strengths and limitations

This study, implemented through a triple-blind design, adopted strict inclusion criteria. We confined the period post stroke to between 6 months and 5 years, and did not include people taking medications that may have the potential to influence cortical activity. While this made recruitment challenging, it partially mitigated the potential influence of confounding factors. Necessarily however, these may remain influential when a small cohort is involved. Additionally, we established through in-depth, one-on-one interviews with each participant, a deep understanding of each stroke survivor’s perceptions of the training program.

There are however, some limitations to consider. While the attempt was made to couple the delivery of NIBS to the progression of each voluntary movement, it was not possible to time this precisely for each reach attempt. Specifically, the onset of tRNS was not yoked directly to the onset of electromyography – for example. In addition, the offset of tRNS was determined by a fixed 5-s interval, rather than triggered by a defined feature of the evolving movement kinematics. In future investigations the timing of tRNS delivery could be linked more directly to the preparation or execution phases of each movement attempt. The sample size employed in the present study was also rather limited. In order to more effectively ascertain the relationships that may exist between the structural integrity of motor output pathways and the potential for recovery of upper limb function in severely impaired stroke survivors, a larger sample – representative of the target population, would be preferred.

## Conclusion

This study highlights that combined interventions that exploit motor learning principles, enable repetitive practice, and seek to enhance cortical drive are feasible and embraced enthusiastically by stroke survivors. Supporting active engagement in movement training in even the most severe stroke survivors has direct benefits for the stroke survivor in terms of enhancing motor recovery and maintaining hope. It also has a positive impact upon carers, who often play a critical role in encouraging and promoting the use of such interventions within the home and community.

## Abbreviations

AI: Asymmetry index
CST: Corticospinal tract
FA: Fractional anisotropy
M1: Primary motor cortex
MAS6: Motor activity scale item 6 upper arm function
MRI: Magnetic resonance imaging
NIBS: Non-invasive brain stimulation
OT-stim: Outcome-triggered electrical stimulation
QoL: Quality of life
REACH: Rating of everyday arm use in the community and home
TMS: Transcranial magnetic stimulation
tRNS: Transcranial random noise stimulation
